# Segmentation of ADPKD Computed Tomography Images with Deep Learning Approach for Predicting Total Kidney Volume

**DOI:** 10.3390/biomedicines13020263

**Published:** 2025-01-22

**Authors:** Ting-Wen Sheng, Djeane Debora Onthoni, Pushpanjali Gupta, Tsong-Hai Lee, Prasan Kumar Sahoo

**Affiliations:** 1 Department of Medical Imaging and Intervention, New Taipei Municipal TuCheng Hospital, Chang Gung Medical Foundation, New Taipei City 236017, Taiwan; steven.sheng@gmail.com; 2 Department of Computer Science and Information Engineering, Chang Gung University, Guishan, Taoyuan 33302, Taiwan; d0421008@cgu.edu.tw (D.D.O.); d0521006@cgu.edu.tw (P.G.); 3 Department of Neurology, Chang Gung Memorial Hospital, Linkou Medical Center, Guishan, Taoyuan 333423, Taiwan; thlee@cgmh.org.tw

**Keywords:** polycystic kidney disease, total kidney volume, non-contrast computed tomography, contrast computed tomography, deep learning, segmentation, localization

## Abstract

**Background:** Total Kidney Volume (TKV) is widely used globally to predict the progressive loss of renal function in patients with Autosomal Dominant Polycystic Kidney Disease (ADPKD). Typically, TKV is calculated using Computed Tomography (CT) images by manually locating, delineating, and segmenting the ADPKD kidneys. However, manual localization and segmentation are tedious, time-consuming tasks and are prone to human error. Specifically, there is a lack of studies that focus on CT modality variation. **Methods:** In contrast, our work develops a step-by-step framework, which robustly handles both Non-enhanced Computed Tomography (NCCT) and Contrast-enhanced Computed Tomography (CCT) images, ensuring balanced sample utilization and consistent performance across modalities. To achieve this, Artificial Intelligence (AI)-enabled localization and segmentation models are proposed for estimating TKV, which is designed to work robustly on both NCCT and Contrast-Computed Tomography (CCT) images. These AI-based models incorporate various image preprocessing techniques, including dilation and global thresholding, combined with Deep Learning (DL) approaches such as the adapted Single Shot Detector (SSD), Inception V2, and DeepLab V3+. **Results:** The experimental results demonstrate that the proposed AI-based models outperform other DL architectures, achieving a mean Average Precision (mAP) of 95% for automatic localization, a mean Intersection over Union (mIoU) of 92% for segmentation, and a mean $R^{2}$ score of 97% for TKV estimation. **Conclusions:** These results clearly indicate that the proposed AI-based models can robustly localize and segment ADPKD kidneys and estimate TKV using both NCCT and CCT images.

## 1. Introduction

Kidney or renal disease can gradually harm the human body by impairing essential renal functions such as filtration, re-absorption, secretion, and excretion. If this condition worsens, the kidney may fail, leading to Chronic Kidney Disease (CKD). Globally, the mortality rate of CKD increased 41.5%, resulting in a total of 1.2 million deaths [1]. According to the Ministry of Health and Welfare (MHW) in Taiwan, nephritis, nephrotic syndrome, and nephrosis were the ninth leading cause of death in 2018 [2]. Autosomal Dominant Polycystic Kidney Disease (ADPKD) ranks as the fourth leading cause of CKD worldwide [3]. The occurrence of ADPKD is primarily attributed to genetic abnormalities that are often inherited from parents. Typically, ADPKD begins to develop asymptomatically in both kidneys. For this reason, its progression is often observed in middle to late adulthood. Thus, it is crucial to predict the progressive loss of renal function at an early stage.

Glomerular Filtration Rate (GFR) [4] is recognized as an important biomarker for predicting the progressive loss of renal function. GFR is measured using blood tests by analyzing changes in serum creatinine levels and estimating GFR (eGFR) values. However, studies have shown that GFR measurements do not reflect changes in serum creatine levels until around the fourth or fifth decade of life [5]. As a result, Total Kidney Volume (TKV) has been included as a second key biomarker alongside GFR. TKV can be calculated using commonly available medical imaging techniques such as Magnetic Resonance Imaging (MRI) and Computed Tomography (CT). Both techniques provide images in three planes, Axial (Transverse), Coronal, and Sagittal, which are stored in the Picture Archiving and Communication System (PACS) in Digital Imaging and Communication in Medicine (DICOM) format. These images typically consist of multiple slices (e.g., ≈100–200 slices). However, MRI is known for being costly and time-consuming. In contrast, CT is a faster and more cost-effective technique, making it highly preferable. CT imaging is categorized into two types: Contrast-enhanced Computed Tomography (CCT) and Non-enhanced Computed Tomography (NCCT). The term “Contrast” refers to a contrast material injected into the patient’s body, which enhances the visibility of specific organs under investigation. However, CCT is not always feasible for ADPKD patients due to the potential side effects of the injected contrast material. As a result, NCCT, which does not require the use of contrast material, is considered as the most practical and widely available medical imaging technique for ADPKD patients, even though some organs may be more difficult to observe.

TKV calculation on CT involves localization and segmentation tasks, which require experienced radiologists to manually localize and segment the kidneys by outlining them slice by slice in the patient’s CT data. Several conventional methods have been applied to this process, including Polyline tracing [6], Livewire [7], Freehand drawing [8], Stereology [9], Mid-Slice [10], and Ellipsoid [11]. However, these methods are reported to be labor-intensive, time-consuming, and prone to human error [12]. For instance, Polyline tracing requires ≈30 min, Livewire takes ≈20–26 min, and Freehand drawing demands 8 min for a single kidney. Moreover, methods like Stereology, Mid-Slice, and Ellipsoid rely on specific values, such as the total number of grids, mid-slice, length, width, and depth, which must be derived using specialized software such as ImageJ [6], OsiriX [8], and others. Despite these tools, the accuracy and efficiency of TKV calculation through localization and segmentation largely depend on a radiologist’s experience and expertise.

To optimize TKV calculation, an automatic segmentation model has been developed using a Computer-Aided Diagnosis (CAD) approach, which is based on Image Preprocessing (IP). A semi-automated segmentation method utilizing IP has been reported, where a T2-weighted MRI was employed to design an ADPKD segmentation model [13]. The method relies on active contours and sub-voxel morphology and considers data from 17 patients. Similarly, an automated segmentation approach based on IP was designed, using a Spatial Prior Probability Map (SPPM) and Propagated Shape Constraint (PSC) techniques, by employing T2-weighted MRI data from 60 patients [14]. However, the IP-based approach has limitations, particularly in terms of low accuracy, which can result in a high error rate. This issue arises from the manual extraction of features, where the low quality of extracted features negatively impacts the performance and accuracy of the segmentation model.

To address these limitations, Artificial Intelligence (AI) techniques have rapidly evolved into two major approaches: Machine Learning (ML) and Deep Learning (DL) [15,16]. Initially, ML techniques were employed to enhance the localization and segmentation capabilities of IP-based approaches. For example, a segmentation method using geodesic distance volume and Random Forest (RF) algorithms was developed to segment ADPKD kidneys on 55 CCT image datasets [17]. Similarly, a preliminary study on 20 NCCT image datasets applied Histogram and K-means algorithms for ADPKD kidney segmentation [18]. Although various ML techniques addressed some limitations of IP-based approaches, their effectiveness heavily depends on the quantity and quality of the training data. The high variability in the shape, intensity, and size of ADPKD kidneys makes small training datasets insufficient for developing robust segmentation models. Additionally, ML techniques rely on optimal handcrafted feature extraction, which can be challenging to achieve consistently. In recent years, DL has achieved tremendous success in handling complex medical image data. Unlike ML, DL introduces automatic feature extraction through the Convolution Neural Network (CNN) methodologies, eliminating the need for handcrafted features. Several DL-based approaches have been designed for image segmentation tasks in ADPKD, utilizing a variety of medical imaging datasets and architectures. For example, Fully Convolutional Network (FCN) and 244 CCT [19], AlexNet and 448 CCT [20], U-Net and 2000 T2-weighted MRI [21], U-Net and 3D T2-weighted [22], FCN and 22 NCCT [23], Region-based CNN (R-CNN) and 32 T2-weighted MRI [24], CNN and 526 T2-weighted MRI [25], Volumetric Medical Image Segmentation (V-Net) with 182 NCCT and 32 CCT [26], and multiple architectures such as FCN, Unet, SegNet, Deeplab, and pspNet and breast 309 Ultrasound image [27].

Based on the existing segmentation techniques, T2-weighted MRI [28] and CCT image data are the most commonly utilized medical imaging modalities for designing automated segmentation models of ADPKD, which use IP, ML, and DL approaches. While two existing ADPKD segmentation methods have involved 20 [24] and 22 [23] NCCT image datasets, the total number of training samples is insufficient for establishing the robustness of the segmentation models. Similarly, 182 NCCT and 32 CCT datasets have been utilized for volumetric analysis [26]. However, the imbalance in the number of NCCT and CCT cases makes it challenging to validate the robustness of the developed models for both modalities.

**Figure 1:**
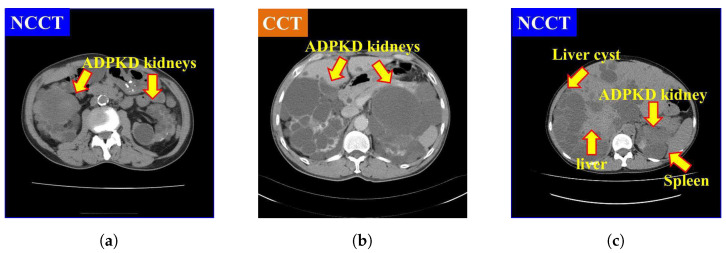
Comparison of NCCT and CCT for ADPKD kidneys: (**a**) ADPKD kidneys on NCCT. (**b**) ADPKD kidneys on CCT. (**c**) ADPKD kidney with adjacent organs such as liver and spleen, alongside liver cyst.

It is also found that NCCT images pose significant challenges for localization and segmentation due to several factors. First, the intensity of NCCT images, as shown in Figure 1a, is lower as compared to the CCT images, as illustrated in Figure 1b. Second, the intensity of liver cysts is similar to that of ADPKD kidneys, as shown in Figure 1c. Third, the intensity of ADPKD kidneys can be similar to adjacent organs such as the liver and spleen, as depicted in Figure 1c. This similarity makes it difficult to differentiate the boundaries of the ADPKD kidneys from neighboring organs during delineation. These limitations of NCCT in capturing detailed structural and functional characteristics of the kidneys can make tasks such as localization, segmentation, and TKV estimations difficult to perform accurately. As ADPKD is a progressive disease, cysts are often smaller and fewer in number during its early stage, which makes diagnosis through NCCT challenging. This difficulty arises because NCCT has limitations in accurately detecting and calculating the total number of cysts in the kidneys [29]. This is crucial in clinical practice as early diagnosis of ADPKD can lead to better patient prognosis. Therefore, incorporating NCCT and CCT for kidney localization, segmentation, and TKV estimation, using AI-based methods, can enhance diagnostic accuracy, improve early risk predictions, and ultimately optimize treatment strategies for better outcomes.

Furthermore, a review of existing studies reveals a lack of investigations into developing an end-to-end model that integrates localization, segmentation, and TKV estimation while addressing the imbalance between NCCT and CCT cases. These challenges have motivated us to design an integrated, robust end-to-end model for ADPKD kidney localization, segmentation, and TKV estimation, which performs effectively on both NCCT and CCT.

In this paper, we propose an automatic approach for the localization, segmentation, and TKV estimation of ADPKD, using a balanced dataset of 100 NCCT and 100 CCT images. Our methodology integrates IP techniques and state-of-the-art DL architectures. Specifically, we adopted the Single Shot Detector (SSD) Inception V2 for the localization model, DeepLab V3+ Xception65 for the segmentation model, and a Decision Tree Regression (DTR) ML model for the TKV estimation model. The main contributions of this paper are summarized as follows:

- Design an automatic localization and segmentation model for ADPKD kidneys, which can effectively work with both NCCT and CCT image data.
- Develop a TKV estimation model, utilizing the outputs of the derived segmentation model.
- Facilitate radiologists’ work by providing automated ADPKD localization, segmentation, and TKV estimation models, thereby reducing the labor involved in analyzing the progressive loss of renal function.

This paper is organized as follows. Section 1.1 reviews existing works related to ADPKD localization and segmentation. Section 2 presents the description of the proposed methods. Section 3 describes the experimental setup and results. Section 4 provides the discussion, while Section 5 concludes the work.

### 1.1. Related Work

This section discusses the evolution of state-of-the-art methodologies for ADPKD kidney localization and segmentation. These methodologies can be categorized into two main groups: without AI (Section 1.1.1), which includes traditional methods and IP-based approaches, and with AI (Section 1.1.2), which encompasses ML and DL approaches.

#### 1.1.1. Without Artificial Intelligence

In clinical practice, traditional methods such as Polyline tracing [6], Livewire [7], Freehand drawing [8], Stereology [9], Mid-Slice [10], and Ellipsoid [11], have been used to delineate and segment the ADPKD kidneys. However, the accuracy of these methods heavily relies on the radiologist’s expertise in manually delineating the kidneys. As a result, high error rates in ADPKD kidney localization and segmentation are inevitable, which can lead to inaccurate TKV calculation. To address the limitations of these traditional methods, IP techniques have been utilized [13,14]. Due to the challenges in delineating ADPKD kidneys on T2-weighted MRI, active contours and sub-voxel morphology were used to automate the delineation process [13]. Specifically, Geodesic Active Contours, Region Competition techniques, and Bridge Burner algorithms were used for the sub-voxel morphology. In [14], instead of using shape model, which are not well-suited for the variable shapes of ADPKD kidneys, a Spatial Prior Probability Map (SPPM) was applied. The process included three steps: SPPM construction, region mapping, and boundary refinement, with the results compared to manual segmentation [14]. While IP-based approaches mitigate some of the challenges of traditional methods, their performance still depends on the quantity and quality of training data. The non-uniform morphology and intensity of ADPKD kidneys make it difficult to build robust localization and segmentation models with insufficient training data. Additionally, IP-based approaches often require handcrafted features, which can be influenced by the radiologist’s expertise in extracting relevant features from the highly variable ADPKD kidney image.

#### 1.1.2. With Artificial Intelligence

To fully automate ADPKD kidney localization and segmentation, the used of AI has rapidly increased. In the realm of ML, the authors [17] applied RF and geodesic distance volume on mid-slice CCT images. Before generating the forest training, feature selection was performed by selecting box features, represented as a single vector with 11 elements. In a preliminary study using a small number of NCCT cases, the authors proposed the first segmentation model for ADPKD kidneys using Histogram analysis, and K-means clustering [18]. By applying ML algorithms, the performance of ADPKD kidney localization and segmentation models can be more accurate compared to IP-based approaches. This is because the model is designed through a learning process using extracted features. However, the performance of the derived ML model depends on the feature extraction and selection methods. If inappropriate features are selected, the localization and segmentation accuracy will be poor. Moreover, during feature extraction and selection, important features might be overlooked. To overcome the limitations of handcrafted feature extraction in IP and ML approaches, DL is increasingly utilized. DL automates the feature extraction process, which is otherwise manually performed in traditional methods. For example, in [19], the authors designed an ADPKD segmentation model using FCN with a Visual Geometry Group (VGG-16) backbone on CCT images. Due to the presence of liver cysts, the localization and segmentation model could potentially overestimate TKV. To address this, the authors [20] proposed a method using AlexNet architecture and Marginal Space Learning (MSL) to improve ADPKD kidney localization and segmentation. Their method classified patches into two classes: abdomen and kidney localization. However, the division of predefined abdomen classes was not analyzed, which could affect the optimal range values derived from the model. The kidney localization model in their approach was created by manually cropping the kidney area and then dividing the cropped images into positive and negative patches. In contrast, the authors [21] designed an ADPKD segmentation model using the U-Net architecture on T2-weighted MRI. They introduced a multi-observer concept, where several iterations were required to find the optimal networks. The final segmentation result was determined by applying a voting scheme among the optimal networks. While this method is robust, it requires a time-consuming training phase.

The U-Net architecture has gained widespread popularity and has been applied in various studies using 2D MRI and 3D CT imaging [30,31,32]. However, 2D segmentation using MRI is limited by the small number of samples used in the architectures [30,31]. In contrast, the authors employed NCCT and CCT for 3D segmentation [32]. Nevertheless, the proportion of NCCT and CCT used in their experiments is imbalanced, with approximately 60% allocated to NCCT and 30% to CCT. Using NCCT image data, an ADPKD kidney segmentation model was developed with an IP technique, where the kidney area was manually cropped, and FCN architecture was applied with two data augmentation techniques, rotation and scaling [23]. However, the proposed method was based on a limited number of NCCT images. For ADPKD volumetry, 182 NCCT and 32 CCT image data were used with V-Net [26]. However, to ensure the robustness of the derived ADPKD kidney segmentation model, balanced data from both CT types is required. To solve this limitation, in this paper, we propose an automatic ADPKD localization and segmentation model by utilizing a balanced number of NCCT (100 patients) and CCT (i.e., 100 samples) images, specifically from ADPKD patients associated with liver cysts.

## 2. Materials and Methods

**Figure 2:**
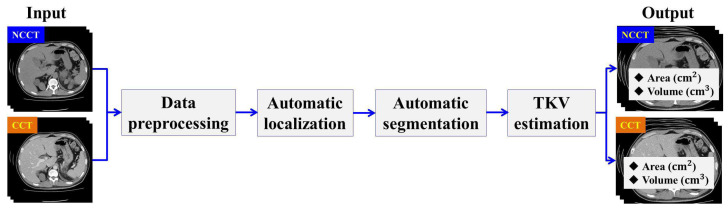
The overview of proposed method workflow.

In this section, we describe the workflow of the proposed method. The designed method consists of four main stages: Data Preprocessing, Automatic ADPKD Kidney Localization, Automatic ADPKD Kidney Segmentation, and TKV Estimation model. An overview of the proposed method workflow is shown in Figure 2.

### 2.1. Data Preprocessing

**Figure 3:**
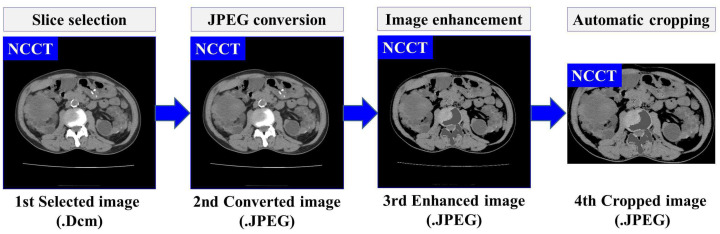
The overview of data preprocessing using NCCT.

Based on our previously developed IP method for ADPKD using CCT [33], we apply four IP procedures to both NCCT and CCT images. First, image selection is performed by excluding images that do not contain both kidneys. Let $R=\{r_{1},r_{2},\ldots ,r_{\left|R\right|}\}$ represent the set of selected raw image data in DICOM format (.Dcm). Each selected image $r_{i}$, where i∈{1,2,…,|R|}, is converted to Joint Photographic Experts Group (JPEG) format using RadiAnt DICOM Viewer software [34]. As shown in Figure 1a,b, both NCCT or CCT images exhibit significantly higher intensity values in the spine compared to other organs, including the ADPKD kidneys. To address this, we apply image enhancement techniques as the third preprocessing step to reduce the intensity variance. Global thresholding is then applied to adjust the coordinates or pixel intensities (x,y) of an image $r_{i}\left(x,y\right)$. The image enhancement operation is expressed in Equation (1).

(1) $${\tilde{r}}_{i}\left(x,y\right)=\left\{\begin{array}{ccccc} Max & \mathrm{if} & r_{i}\left(x,y\right) & > & T_{max}\\ 0 & \mathrm{if} & r_{i}\left(x,y\right) & < & T_{min} \end{array}\right\}$$

where ${\tilde{r}}_{i}\left(x,y\right)$ represents the enhanced image, $T_{min}$ and $T_{max}$ are the minimum and maximum thresholds, respectively, and Max refers to a user-defined maximum intensity. These threshold values are determined based on empirical studies conducted separately for each case in both NCCT and CCT, with the values being consistent within each modality. Additionally, to avoid bias in the image, an automatic cropping mechanism is designed. This mechanism works by cropping the full black pixel intensity $\left({\tilde{r}}_{i}\left(x,y\right)=0\right)$ that surrounds the abdomen cavity. To achieve this, dilation morphology [35] and contour [36] techniques are applied. The dilation morphology operation for each input-enhanced image ${\tilde{r}}_{i}$ can be computed as follows:

(2) $$DILATION\left(B⊕S\right)=\underset{s\in S}{⋃}B_{s}$$

where *B* is the input binary image with a kernel size S=4×4, and $B_{s}$ represents the translation of image *B* by *s*. The hierarchical contour technique is adopted to identify the maximum contour area in the image. Then, a rectangular shape Rec is drawn around the maximum area with coordinates Rec=[y:y+h,x:x+w], where h,w refer to the height and width, respectively. Afterward, the corresponding rectangular area Rec is cropped. The result of this preprocessing is the enhanced images $\tilde{r_{i}}$. Furthermore, a set of preprocessed images $\tilde{R}$, $\tilde{R}=\left\{{\tilde{r}}_{1},{\tilde{r}}_{2},...,{\tilde{r}}_{|\tilde{R}|}\right\}$ is obtained, which will be used as the training input for developing the automatic localization and segmentation models. The overall flow and an example of proposed data preprocessing steps are illustrated in Figure 3.

### 2.2. Automatic ADPKD Kidney Localization

**Figure 4:**
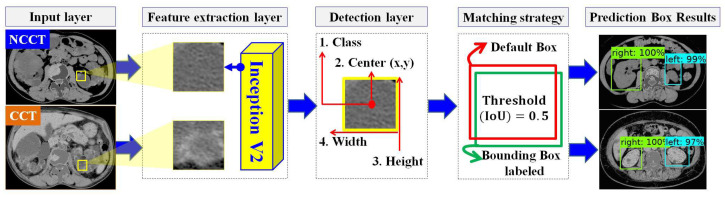
The overview of automatic ADPKD kidney localization model.

In designing the automatic ADPKD kidney localization model, we extend and improve upon our previous work [33]. Based on the results of our prior experiments, we demonstrated that one of the one-stage detector algorithms, SSD Inception V2, outperforms other two-stage and one-stage detectors. Therefore, in this work, we select SSD Inception V2 again, with more extensive experiments involving 100 NCCT and 100 CCT. SSD Inception V2 consists of two main layers: the feature map extraction layer and the detection layer, as shown in Figure 4. Initially, the preprocessed input image $\tilde{r_{i}}$ (either NCCT or CCT) is labeled by drawing a set of bounding boxes $Box=\{b_{left},b_{right}\}$, where each image contains a total number of bounding boxes, 0<|Box|≤2. After labeling the bounding boxes using Labellmg software v1.8.4, each image has an associated label file $f_{j}$, which contains a set of tuples. Each tuple comprises the bounding box coordinates $C_{oor}$, $C_{oor}=\left[y:y+h,x:x+w\right]$ and the respective class $C_{ls}$, where $C_{ls}=\left\{c_{left},c_{right}\right\}$. The label file $f_{j}$ is thus represented as $f_{j}=\left\{<C_{oor}^{1},C_{ls}^{1}>,...,<C_{oor}^{\left|Box\right|},C_{ls}^{\left|Box\right|}>\right\}$. The pair with the input image and label $\left(\tilde{r_{i}},f_{j}\right)$ is then fed into the feature map extraction layer using the Inception V2 backbone. This layer extracts features through 16 convolution layers and Rectified Linear Unit (ReLU) activation functions. The extracted feature maps are subsequently forwarded to the detection layer, where four components need to be detected: the total number of classes *C*, the center of the bounding box $t_{x,y}$, the width *w*, and the height *h*. To achieve this, a total of |N| default boxes, $N=\{d_{1},d_{2},...,d_{\left|N\right|}\}$, with different aspect ratios $r=\{1,2,3,\frac{1}{2},\frac{1}{3}\}$ and scales sl, are required. The determination of different scales is shown in Equation (3).

(3) $$sl=s_{min}+\frac{s_{max}-s_{min}}{|f|-1}\left(i-1\right),i\in \left[1,|f|\right]$$

where $s_{min}=0.2$ is the scale for the lowest layer, $s_{max}=0.95$ is the scale for the highest layer, and |f| refers to the total number of extracted feature maps. A matching procedure *M* is performed by comparing the coordinates and corresponding class labels in the label file $f_{j}$ with each default box $d_{m}$, m={1,2,...,|N|}. The matching procedure is outlined in Equation (4).

(4) $$M\left(f_{j},d_{m}\right)=\left(\begin{array}{cccc} f_{j}[y: & y+h, & x: & x+w]\\ d_{m}[y: & y+h, & x: & x+w] \end{array}\right)$$

The matching procedure is based on the best Jaccard index or Intersection over Union (IoU). As a result, the localization outputs are generated, including the predicted bounding box $B^{m}$, m∈Box, the predicted class $c^{n}$, $n\in C_{ls}$, and the localization confidence score $L_{score}$.

### 2.3. Automatic ADPKD Kidney Segmentation

**Figure 5:**
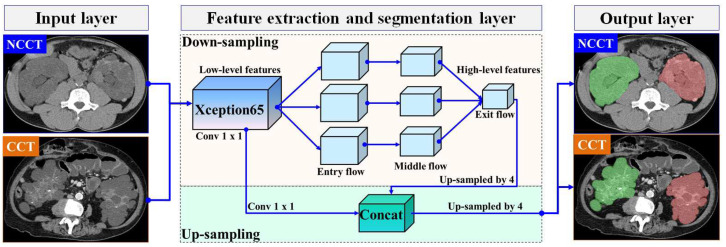
The overview of automatic ADPKD kidney segmentation model: Green represents the segmented right kidney, red represents the segmented left kidney, white indicates high-density areas (e.g., spine), and gray represents low-density areas (e.g., surrounding organs and soft tissues).

In this section, we present our proposed automatic ADPKD segmentation model for NCCT and CCT image datasets. The segmentation problem is approached as a semantic segmentation task, focusing on two main Regions of Interest (RoIs): the left and right kidneys. To solve this semantic segmentation problem, we adopt the DeepLab V3+ architecture [37], an enhanced version of DeepLab V3 [38], developed by Google. Although many architectures are available for segmentation models, such as U-Net, we chose to adopt DeepLab V3+ due to our objective of segmenting the entire kidney, which contains multiple cysts of varying sizes and structures. U-Net is known for its high accuracy in segmenting large areas. However, it suffers from jagged boundaries and the islanding phenomenon, where its symmetric skip connections, which combine high- and low-level features, can locate regions but result in jagged cyst boundaries and potentially overlook overlapping cysts [39]. Therefore, we selected DeepLab V3+ as it is better suited for capturing entire kidneys containing cysts of varying sizes and fine details, thanks to its Atrous Spatial Pyramid Pooling (ASPP), which are crucial for accurately estimating TKV. DeepLab V3+ introduces three key concepts: depth-wise separable convolution with dilation (also known as dilated convolution), Atrous Spatial Pyramid Pooling (ASPP) with image-level features, and batch normalization. In our proposed ADPKD segmentation model, the DeepLab V3+ network is organized into three main layers: the input layer, the feature extraction and segmentation layer, and the output layer. An overview of the proposed automatic ADPKD kidney segmentation model is shown in Figure 5.

Similar to the automatic localization model, with the exception of the third preprocessing step (i.e., image enhancement), the preprocessed image $\tilde{r_{i}}$ (either NCCT or CCT) and the corresponding mask image $m_{j}$ are treated as pairs for the input layer $\left(\tilde{r_{i}},m_{j}\right)$. The mask image is generated using the open-source software LabelMe v3.16.1, where each mask image contains at least one mask region, denoted as $m_{j}^{k}$, where k∈{right,left}. In [37], the entire image is used as input, where the crop size corresponds to the maximum size of the input. Therefore, we set the input size to 513×513.

A pair of inputs is fed into the feature extraction and segmentation layer. In the feature extraction layer, we adopt Xception65 as the backbone for feature extraction in our segmentation model, which consists of 65 network layers. Xception introduces two main ideas: pointwise convolution followed by deepwise convolution where the Max Pooling operation is replaced with separable (conv2d) with a stride of 2 and a ReLU activation function [40]. The extracted feature maps are then fed into the Atrous Spatial Pyramid Pooling (ASPP) layer, utilizing atrous rates a={6,12,18,16}, along with batch normalization. Finally, the extracted features are combined using a 1 × 1 convolution. At the end of this process, the prediction is made by calculating the probability of the input vector *i* using the Softmax function, as shown below:

(5) $$Softmax\left(i\right)=\frac{exp(i)}{\sum_{j=1}^{\eta }exp\left(j\right)}$$

where η is the total input vector from the exit flow section, and exp(i) represents the exponentiation of the particular input vector i,j∈η. To obtain a high-level feature map, an up-sampling procedure is executed on the extracted feature maps. We use a stride of 4 for the up-sampled decoder output. As a result, the outputs from the low-level extracted features are concatenated with high-level extracted features. Finally, in the output layer, the segmented areas are generated, which correspond to either the left kidney, the right kidney, or both kidneys.

### 2.4. TKV Estimation Model

To finalize the end-to-end proposed method, we analyze and design the TKV estimation model, which consists of three main stages: feature extraction, numerical data preprocessing, and TKV estimation using the DTR ML algorithm. We select the DTR model because it is efficient on large numerical datasets and effectively handles non-linearity. Additionally, DTR is a simpler algorithm compared to other regression ML algorithms. In the first stage, we extract features from two sources: the ground truth provided by radiologists (i.e., CSV format) and the segmented results derived from the AI model. Let $G=\{g_{1},g2,...,g_{\left|G\right|}\}$ represent the set of extracted features from ground truth, and let $h_{i}$ denote the segmented area derived from the automatic ADPKD kidney segmentation result. An element of $g_{j}$, where j∈1,2,...,|G|, represents features such as the mean Hounsfield Unit (HU), area, and volume of kidneys. The $h_{i}$ values correspond to the contour segmented areas of the left and right kidneys, where i∈{left,right}. To calculate the segmented contour areas, we detect the contours of the segmented kidneys by identifying points surrounding the objects. Using these detected points, we apply the Green Formula to calculate the areas within the contours as described in [41]. The final set of features for TKV ($F_{tkv}$) is obtained as the union of the features from the ground truth and segmented contour areas, $F_{tkv}=\left\{G\cup H\right\}$, where $F_{tkv}=\left\{\varepsilon_{1},\varepsilon_{2},...,\varepsilon_{|F_{tkv}|}\right\}$. For each extracted feature $\varepsilon_{r}$, $r=\{1,2,...,|F_{tkv}|\}$, we apply numerical preprocessing by normalizing the data points $d^{r}$, $d\in \varepsilon_{r}$ using min–max normalization [42], as shown in Equation (6).

(6) $$\tilde{d^{r}}=\frac{d^{r}d_{min}^{r}}{d_{max}^{r}-d_{min}^{r}}$$

where $d_{min}^{r}$ and $d_{max}^{r}$ are the minimum min() and maximum values max() observed from all data points *D* corresponding to a particular extracted feature *r*, respectively. We apply the DTR model from the Sckit-learn machine learning framework. The model is built using the Classification And Regression Tree (CART) algorithms. Given the set of normalized features ${\tilde{F}}_{tkv}$, a regression tree is generated. To evaluate the quality of feature splits, the Mean Squared Error (MSE) is computed, as described in [43], and it is shown in Equation (7):

(7) $$MSE=\frac{1}{D}\sum_{d=1}^{D}{\left(x_{d}-{\bar{x}}_{d}\right)}^{2}$$

where $x_{d}$ and ${\bar{x}}_{d}$ are the observed data points and predicted data points, respectively.

## 3. Results

In this section, we provide a detailed discussion of the experiment, which includes the following components: the Dataset, the Experimental Setup, the Evaluation Metrics, the Results for ADPKD kidney localization, segmentation and TKV estimation models on NCCT and CCT images.

### 3.1. Dataset

To design a robust ADPKD localization, segmentation, and TKV estimation model, we collected a dataset of 200 CT scans, comprising 100 NCCT and 100 CCT scans, all associated with liver cysts. These 200 CT scans were obtained from 97 ADPKD patients. As shown in the demographic Table 1, the majority of our sample is male with an average age of 55 and a mean TKV of 2734.33 cm^3^. The dataset includes a total of 17,836 slices, with 8849 NCCT slices and 8987 CCT slices. These images were collected from the PACS system of Linkou Chang Gung Memorial Hospital between 2003 and 2019. The Institutional Review Board (IRB) of the Chang Gung Medical Foundation, Taipei, Taiwan, approved this study (IRB No. 201701583B0C501). The CT slices have a thickness and interval of 5 mm each. The images are in DICOM format with a window level of 35 HU and a window width of 350 HU. The raw CT images have a dimension of 512 × 512 pixels. The collected CT raw image data were annotated by two radiologists with 10 years of experience. The annotations provided in the form of annotated images and CSV files serve as the ground truth for the dataset. Based on this ground truth, the number of usable CT raw image data was reduced to 8618, consisting of 4352 NCCT and 4266 CCT images. For model training and testing, the dataset was split into 80% training and 20% testing sets. This results in 3482 training images (i.e., 80 NCCT scans) and 870 testing images (i.e., 20 NCCT scans), and 3413 training images (i.e., 80 CCT scans) and 853 testing images (i.e., 20 CCT scans). It is important to note that the training images are not included in the testing set. For the training set, we applied k-fold cross validation |k|=5. The training set was divided randomly into approximately equal-sized subsets. During each of the 5 rounds of training, one subset $k_{d}$, where d={1,2,...,|K|}, was used as the validation set, while the remaining |k−1| subsets were used for training.

### 3.2. Experimental Setup

Our experiments are conducted using the OpenCV v3.2.0 data preprocessing framework, TensorFlow-GPU v1.12 DL framework, and Scikit-learn v0.19.2 ML framework. The interface for all frameworks is implemented in Python v3.6.7, along with several other supported Python libraries, including Pandas v1.0.5, Numpy v1.19.1, Matplotlib v3.3.0, Pillow v7.2.0, osmnx v0.15, lxml v4.2.1, imageio v2.5.0, Urllib3 v1.22, Sys v3.6.9. All software is available for access and installation from this Python Package Index (PyPI) repository [44]. These frameworks are running on an Ubuntu 18.04.3 operating system with the following hardware specifications: GPU TITAN RTX 24 GB × 4 and 256 GB memory.

### 3.3. Evaluation Metrics

We evaluate our ADPKD kidney localization and segmentation models using four common evaluation metrics: Accuracy (ACC) (Equation (8)), Precision (PR) (Equation (9)), Recall/Sensitivity (RE) (Equation (10)), and Dice-score (DS) (Equation (11)). These metrics can be calculated as follows:

(8) $$\mathrm{ACC}=\frac{\mathrm{TP}}{\mathrm{TP}+\mathrm{FN}+\mathrm{FP}}$$

(9) $$\mathrm{PR}=\frac{\mathrm{TP}}{\mathrm{TP}+\mathrm{FP}}$$

(10) $$\mathrm{RE}=\frac{\mathrm{TP}}{\mathrm{TP}+\mathrm{FN}}$$

(11) $$\mathrm{DS}=2\times \frac{\mathrm{PR}\times \mathrm{RE}}{\mathrm{PR}+\mathrm{RE}}$$

where TP refers to True Positive, FP denotes False Positive, FN stands for False Negative, and TN represents True Negative. Moreover, for automatic localization and segmentation, the Intersection over Union (IoU) metric was used by calculating the intersection between predicted and ground truth RoI, divided by their union. We also computed the mean IoU (mIoU), Average Precision (AP), and mean AP (mAP). R-square ($R^{2}$) coefficient was utilized to assess the performance of the regression-based TKV estimation model. The $R^{2}$ was calculated by dividing the Sum of Squares Regression (SSR) by the Sum of Squares Total (SST).

### 3.4. ADPKD Kidney Localization Results

We trained and compared the proposed automatic localization model with well-established object localization architectures such as the original SSD and Faster R-CNN, which have been applied to malignant pulmonary nodule detection [45]. After preprocessing (Section 2.1), the training data were used for training and validation purposes with the designed localization model, SSD MobileNet V1, and Faster R-CNN Inception ResNet V2 across *k* rounds. Similarly, we tested the derived models on an independent testing set to compare and evaluate the robustness of our proposed localization model. The performance results of the designed model on NCCT and CCT were analyzed and presented in two parts: validation set and testing set results.

#### 3.4.1. Validation Set Results on NCCT

By applying k-fold cross-validation, we evaluated the performance of the proposed ADPKD kidney localization model. As shown in Table 2, we compared the performance of our model with other localization architectures in terms of ACC, PR, RE, DS, and mAP. The results show that our proposed method achieves an average of 92% across all metrics. In comparison, SSD MobileNet and Faster R-CNN Inception ResNet V2 achieve 84% and 46%, respectively.

#### 3.4.2. Testing Set Results on NCCT

**Figure 6:**
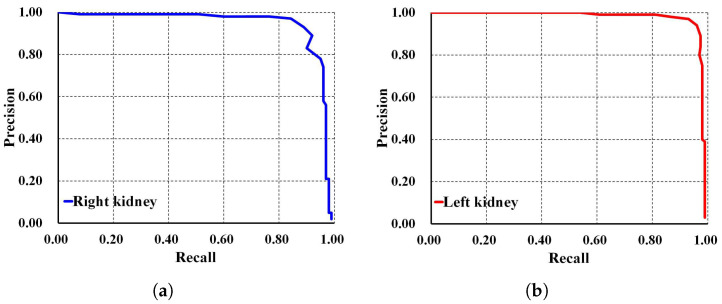
Precision and recall curve on NCCT: (**a**) Right kidney. (**b**) Left kidney.

**Figure 7:**
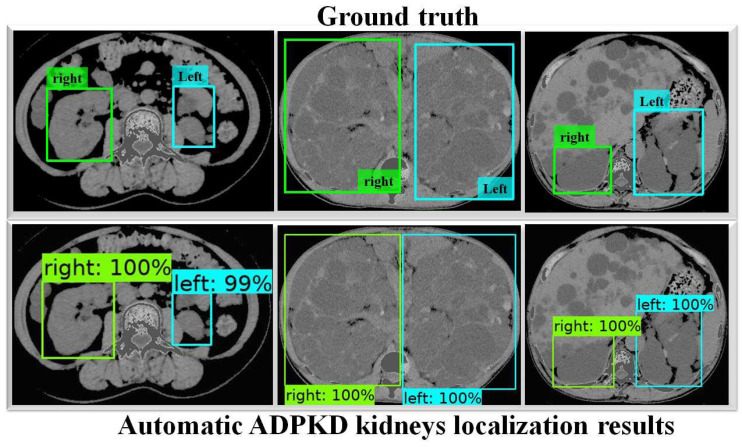
Automatic ADPKD kidney localization results on NCCT.

To thoroughly evaluate the performance of the proposed localization model, we test the derived model using an independent testing set and compare it with other localization architectures. The results show that our derived model outperforms the others with 94%, 96%, 91%, 93%, and 96% for ACC, PR, RE, DS, and mAP, respectively, as shown in Table 3. In comparison, SSD MobileNet V1 and Faster R-CNN Inception ResNet V2 achieved only 89% and 41%, 88% and 42%, 89% and 51%, 89% and 45%, and 90% and 55% for ACC, PR, RE, DS, and mAP, respectively. Notably, our proposed localization model achieves the highest mAP of 96%. The PR and RE curve for the right and left kidneys can be visualized in Figure 6a,b, respectively. Moreover, our ADPKD localization model demonstrates robustness in detecting ADPKD kidneys with varying sizes, shapes, and intensities (including those similar to liver cysts), as shown in Figure 7.

#### 3.4.3. Validation Set Results on CCT

Using the same comparison setup and evaluation metrics, we assess the performance of the derived localization model on CCT. As shown in Table 4, our proposed method demonstrates the ability to localize ADPKD kidneys with 91% of mAP, outperforming SSD MobileNet V1 and Faster R-CNN Inception ResNet V2, which achieve 78% and 51% mAP, respectively.

#### 3.4.4. Testing Set Results on CCT

Using an independent testing set, each localization architecture model showed significant improvement, as shown in Table 5. While SSD MobileNet V1 localizes ADPKD kidneys with 82% ACC, 83% PR, 84% RE, 83% DS, and 86% mAP, our proposed method demonstrates superior performance, achieving 91% ACC, 93% PR, 87% RE, 90% DS, and 94% mAP in localizing ADPKD kidneys.

**Figure 8:**
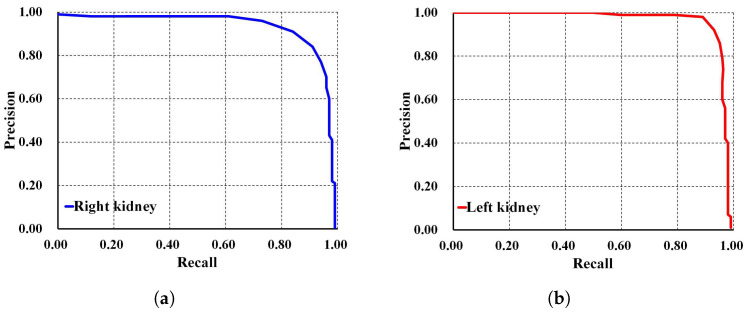
Precision and recall curve on CCT: (**a**) Right kidney. (**b**) Left kidney.

**Figure 9:**
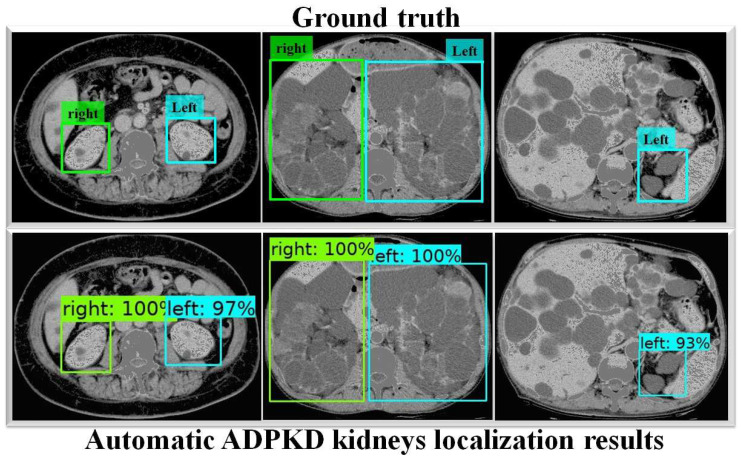
Automatic ADPKD kidney localization results on CCT.

In addition, Faster R-CNN Inception ResNet V2 shows the lowest performances compared to both SSD MobileNet V1 and our proposed method. For a deeper performance analysis, we derive the PR and RE curves for the left and right kidneys based on the highest mAP value of 94%, as shown in Figure 8a,b, respectively. Figure 9 shows the visualization of our automatic ADPKD kidney localization on CCT.

### 3.5. ADPKD Kidney Segmentation Results

To evaluate the performance of our proposed automatic ADPKD kidney segmentation method, we select commonly used semantic segmentation architecture for ADPKD kidney segmentation, such as FCN [19] with the powerful VGG-16 [46] backbone, and the original DeepLab V3+ architecture with MobileNet V2, which has shown the best performance for breast tumor semantic segmentation in state-of-the-art comparison [47]. We train our model, along with the two selected architectures, using the preprocessed training dataset. Training and validation are performed over *k* rounds. We analyze and compare the performance of our model against the other architectures using an independent testing set for *k* rounds of testing. These procedures are applied to both NCCT and CCT, with results presented in two categories: validation set results and testing set results for both NCCT and CCT.

#### 3.5.1. Validation Set Results on NCCT

Table 6 presents a comparison between our method and the FCN with VGG-16 and DeepLab V3+ with MobileNet architectures. It can be observed that our proposed method outperforms the other semantic segmentation architectures, achieving an average of 94% across the PR, RE, DS, and mIoU metrics, with an average Standard Deviation (SD) value of ±0.004. This shows that, over *k* rounds of validation, our method consistently exhibits robust performance in segmenting the ADPKD kidneys, outperforming both the FCN with VGG-16 and DeepLab V3+ with MobileNet V2 architectures.

#### 3.5.2. Testing Set Results on NCCT

**Figure 10:**
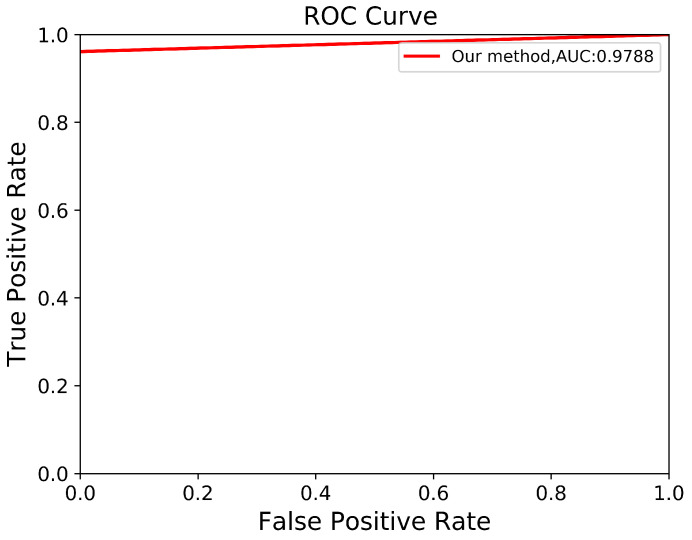
Automatic ADPKD segmentation ROC curve on NCCT.

**Figure 11:**
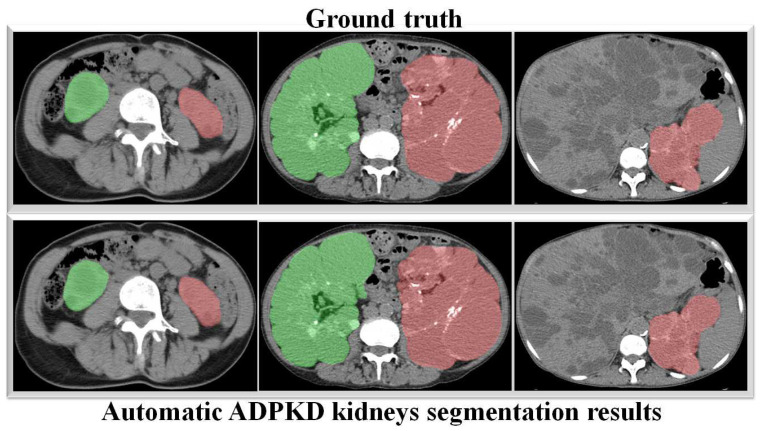
Automatic ADPKD kidney segmentation results on NCCT: Green represents the segmented right kidney, red represents the segmented left kidney, white indicates high-density areas (e.g., spine), and gray represents low-density areas (e.g., surrounding organs and soft tissues).

Through a more in-depth evaluation, we compare the performance of our proposed method with other architectures using the testing set. As shown in Table 7, the performance of our method in terms of mIoU slightly increases from 91% to 92%, with the lowest SD of ±0.002. With an unseen dataset, our method can robustly segment the ADPKD kidneys, achieving 96%, 95%, 95% for PR, RE, and DC, respectively. To support these findings, we plot the Receiver Operating Characteristic (ROC) curve to analyze the trade-off between the True Positive Rate and False Positive Rate. As shown in Figure 10, our method achieves a higher Area under Curve (AUC) = 0.978. Figure 11 displays the comparison between the ground truth and the automatic segmentation results. These results demonstrate that our designed model can segment ADPKD kidneys precisely, even though the kidneys vary in shape and size, and their intensity is similar to adjacent organs and liver cysts.

#### 3.5.3. Validation Set Results on CCT

Similar to the NCCT evaluation, we assess and compare our proposed method with other architectures using CCT. Table 8 shows the experiment results, including the SD. Our proposed segmentation method outperforms other state-of-the-art semantic segmentation architecture, achieving 95%, 95%, 95%, 91% value for PR, RE, DS, and mIoU. The low SD values across all metrics show that our method performs robustly over *k* rounds of validation.

#### 3.5.4. Testing Set Results on CCT

**Figure 12:**
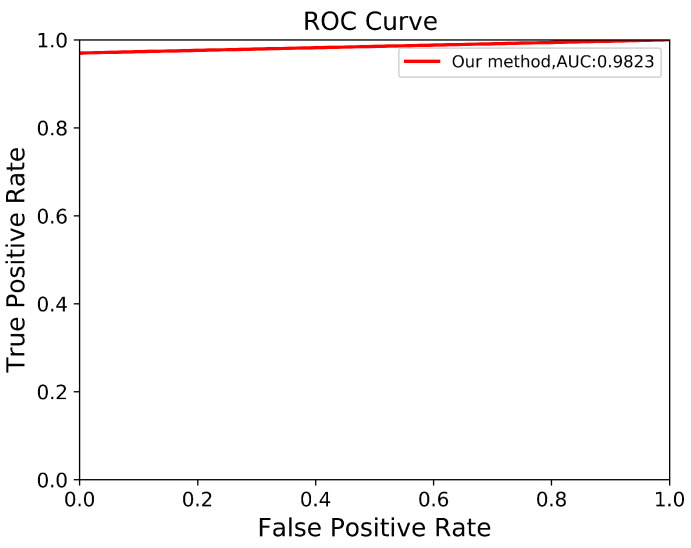
Automatic ADPKD segmentation ROC curve on CCT.

**Figure 13:**
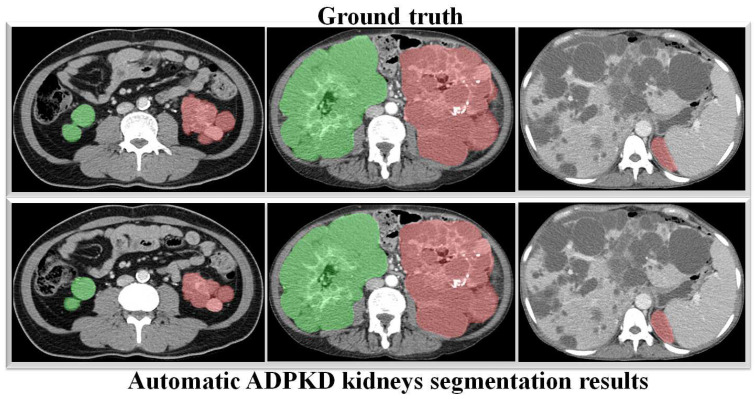
Automatic ADPKD kidney segmentation results on CCT: Green represents the segmented right kidney, red represents the segmented left kidney, white indicates high-density areas (e.g., spine), and gray represents low-density areas (e.g., surrounding organs and soft tissues).

To verify the robustness of our derived method, we evaluate it again using an independent CCT testing set. The evaluation is conducted in the same manner for our model and the other comparison models. As shown in Table 9, the results demonstrate that our model outperforms FCN with VGG-16 and DeepLab V3+ with MobileNet V2. Our model achieves an average of 95% across all metrics for segmenting ADPKD kidneys on CCT, while FCN with VGG-16 and DeepLab V3+ MobileNet only achieve an average of 72% and 79%, respectively. We visualized the performance of our model through an ROC curve, as shown in Figure 12, where our method achieves a high AUC value of 0.982. As shown in Figure 13, these results confirm that our proposed segmentation ADPKD kidneys method can accurately segment kidneys, even in the presence of morphological heterogeneity.

### 3.6. TKV Estimation Results

**Figure 14:**
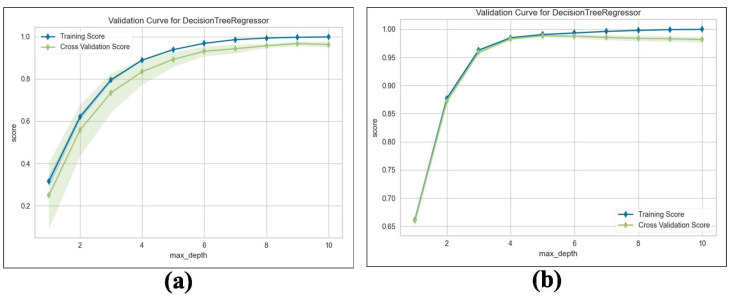
Validation curve for DTR: (**a**) Validation score on NCCT. (**b**) Validation score on CCT.

We train the proposed TKV estimation using the DTR model. We evaluate and compare the performance of the adopted regression model with Linear Regression (LR), measuring the results using an $R^{2}$ score with 5-fold cross validation. Table 10 shows the results obtained from both NCCT and CCT. Our method estimates superior performance, which achieves an average Maximum $R^{2}$ = 98% for predicting TKV based on the given input features compared to 87% for LR. For a deeper analysis, we examine the training and validation scores with varying values of the maximum tree depth hyperparameters. As shown in Figure 14a,b, the proposed method’s training and validation scores improve as the maximum tree depth hyperparameter increases.

## 4. Discussion

Analyzing the progressive loss of renal function in ADPKD patients through medical image data is crucial. Therefore, accurate TKV measurement on NCCT and CCT scans is both essential and challenging. In this paper, we propose an AI-based framework for ADPKD kidney localization, segmentation, and TKV measurement for both NCCT and CCT. The proposed method integrates traditional IP techniques with DL architectures, such as SSD Inception V2 for localization, DeepLab V3+ Xception65 for segmentation, and an ML approach using the DTR algorithm for TKV estimation.

In the first part of this work, we proposed an automatic ADPKD localization model for both NCCT and CCT. The localization model was trained and validated using the training set for over *k* rounds, which was followed by testing on an independent testing set. Based on the evaluation results, our model outperformed SSD MobileNet V1 and Faster R-CNN Inception ResNet V2 architectures, achieving 92% ACC, 94% PR, 89% RE, 95% DS, and 95% mAP. Our proposed localization model demonstrates a higher mAP than previous works such as [24,46], where R-CNN-based detection resulted in a high false positive, leading to low AP = 78% when MRI was used as input. As shown in Figure 7 and Figure 9, our localization model accurately localizes the left kidney (i.e., blue box) and right kidney (i.e., green box) with a high confidence score. Despite the presence of liver cysts, our model successfully localizes the ADPKD kidneys by predicting the bounding boxes, although some parts of adjacent organs, like the liver and spleen, may also be included within the detected box. To address this limitation, we further proposed an automatic ADPKD segmentation model.

The main aim of our second proposed idea is to extract the precise shape of ADPKD kidneys from both NCCT and CCT. Similar to the first localization idea, we trained and validated the segmentation model over *k* rounds and then tested the model using a separate testing set. As shown in Table 7 and Table 9, the proposed model achieved an average of 96% PR, 95% RE, 95% DS, and 92% mIoU on both NCCT and CCT. With an mIoU = 92%, our method outperforms other ADPKD segmentation architectures [24,46], which achieved an IoU of 85% using MRI images. When using CCT as input, our proposed segmentation model achieved a higher DS = 0.96(±0.001) compared to [19], which reported as DS of 0.85(±0.007). These results are reflected in the segmented images shown in Figure 11 and Figure 13 for NCCT and CCT, respectively. It can be observed that our segmentation model robustly segments ADPKD kidneys of varying sizes and shapes, even in the presence of liver cysts. The outputs of this segmentation model are then used to design the TKV estimation model. To complete our proposed idea, we introduce the third component: the TKV estimation model. As discussed in the performance evaluation results, our proposed method using DTR achieved high $R^{2}=98.7\%$ for NCCT and $R^{2}=98.3\%$ for CCT. Using k-fold cross validation, the TKV estimation model achieved $R^{2}$ =96.2% with an SD of 0.01 for NCCT and $R^{2}$ = 98% with an SD of 0.004 for CCT.

One of the limitations of our study is the small dataset size, which can affect the generalization of our models. Although our results show high mIoU, some segmentation errors do occur in certain cases, such as when cysts overlap with neighboring organs or have homogeneous intensity. These errors may impact the subsequent task of TKV estimation, and as a result, inaccurate TKV could affect treatment decisions or patient outcomes. To address the small dataset size and segmentation errors, we plan to conduct future experiments with a large dataset and additional external validation. By doing so, we aim to improve the generalizability and reliability of our models. Despite strong performance and lower sensitivity to outliers compared to other regression algorithms, outliers can still influence how the decision tree splits the data. As a result, the tree may overfit to extreme outliers, causing splits that fail to reflect the true distribution of the data. Consequently, this could result in less precise TKV estimations. In the future, we plan to mitigate the influence of outliers by employing outlier removal techniques.

To make the proposed method feasible in real-world settings, we could integrate the automatic ADPKD kidney localization, segmentation, and TKV estimation into a single, user-friendly desktop-based software pipeline in the future. This software could be installed in hospital systems, allowing clinicians and radiologists to compare their conventional methods with our software. This integration can enhance diagnostic accuracy and decision-making for treatment. Additionally, through this comparison, we can validate our model’s results against conventional methods, ensuring the reliability and accuracy of the developed software.

## 5. Conclusions

In this paper, three key models, an automatic ADPKD localization model, a segmentation model, and a TKV estimation model, which utilized IP methods and DL approaches, were proposed. These models were designed to work robustly on NCCT and CCT images. For IP, we applied techniques such as image enhancement and automatic cropping. For the localization model, we adopted SSD Inception V2; for segmentation, we used DeepLab V3+ Xception65; and for the TKV estimation model, we implemented the DTR model. The experimental results demonstrate that our localization model achieves a mAP of 95%, our segmentation model achieves a mIoU of 92%, and the TKV estimation model reaches an $R^{2}$ of 97%. These results show that our derived models work robustly on both NCCT and CCT images, even when considering challenges such as liver cysts and variations in kidney shape and size. Furthermore, we believe that derived models could significantly assist radiologists in diagnosing the progressive loss of renal function, particularly when working with challenging imaging modalities like NCCT and CCT.

## Figures and Tables

**Table 1:** Demographic information of 200 CT scans from 97 ADPKD patients.

Characteristic		Values
Age (years; Mean ± SD)		55 ± 18.5
Sex (total sample; %)	Male	52 (53.6%)
	Female	45 (46.4%)
TKV (cm^3^; Mean ± SD)		2734.33 ± 2312.45

**Table 2:** Localization results using validation set with k-fold on NCCT, including Accuracy (ACC), Precision (PR), Recall (RE), Dice-score (DS), and mean Average Precision (mAP).

Architecture	ACC	PR	RE	DS	mAP
Our method	0.91 (±0.07)	0.94 (±0.04)	0.9 (±0.05)	0.91 (±0.041)	0.94 (±0.02)
SSD MobileNet V1	0.83 (±0.08)	0.84 (±0.06)	0.85 (±0.05)	0.85 (±0.06)	0.85 (±0.02)
Faster R-CNN Inception ResNet V2	0.4 (±0.02)	0.41 (±0.03)	0.51 (±0.01)	0.44 (±0.02)	0.55 (±0.03)

**Table 3:** Localization results using testing set NCCT, including Accuracy (ACC), Precision (PR), Recall (RE), Dice-score (DS), and mean Average Precision (mAP).

Architecture	ACC	PR	RE	DS	mAP
Our method	0.94 (±0.02)	0.96 (±0.01)	0.91 (±0.01)	0.93 (±0.01)	0.96 (±0.01)
SSD MobileNet V1	0.89 (±0.03)	0.88 (±0.02)	0.89 (±0.01)	0.89 (±0.02)	0.9 (±0.01)
Faster R-CNN Inception ResNet V2	0.41 (±0.01)	0.42 (±0.02)	0.51 (±0.01)	0.45 (±0.01)	0.55 (±0.02)

**Table 4:** Localization results using validation set with k-fold on CCT, including Accuracy (ACC), Precision (PR), Recall (RE), Dice-score (DS), and mean Average Precision (mAP).

Architecture	ACC	PR	RE	DS	mAP
Our method	0.83 (±0.06)	0.88 (±0.04)	0.82 (±0.05)	0.85 (±0.04)	0.91 (±0.03)
SSD MobileNet V1	0.62 (±0.09)	0.78 (±0.06)	0.79 (±0.06)	0.77 (±0.07)	0.78 (±0.07)
Faster R-CNN Inception ResNet V2	0.43 (±0.04)	0.38 (±0.02)	0.5 (±0.03)	0.42 (±0.01)	0.51 (±0.04)

**Table 5:** Localization results using testing set CCT, including Accuracy (ACC), Precision (PR), Recall (RE), Dice-score (DS), and mean Average Precision (mAP).

Architecture	ACC	PR	RE	DS	mAP
Our method	0.91 (±0.03)	0.93 (±0.02)	0.87 (±0.01)	0.9 (±0.01)	0.94 (±0.01)
SSD MobileNet V1	0.82 (±0.08)	0.83 (±0.04)	0.84 (±0.04)	0.83 (±0.05)	0.86 (±0.05)
Faster R-CNN Inception ResNet V2	0.38 (±0.01)	0.39 (±0.02)	0.5 (±0.01)	0.43 (±0.02)	0.51 (±0.01)

**Table 6:** Segmentation results using validation set with k-fold on NCCT, including Precision (PR), Recall (RE), Dice-score (DS), and mean IoU (mIoU).

Architecture	PR	RE	DS	mIoU
Our method	0.96 (±0.004)	0.95 (±0.004)	0.96 (±0.003)	0.91 (±0.007)
Fully Convolution Network with VGG-16	0.73 (±0.3)	0.63 (±0.3)	0.66 (±0.3)	0.63 (±0.1)
DeepLab V3+ with MobileNet V2	0.88 (±0.01)	0.89 (±0.004)	0.88 (±0.007)	0.79 (±0.007)

**Table 7:** Segmentation results using testing set NCCT, including Precision (PR), Recall (RE), Dice-score (DS), and mean IoU (mIoU).

Architecture	PR	RE	DS	mIoU
Our method	0.96 (±0.004)	0.95 (±0.003)	0.95 (±0.002)	0.92 (±0.002)
Fully Convolution Network with VGG-16	0.87 (±0.05)	0.84 (±0.09)	0.84 (±0.08)	0.76 (±0.05)
DeepLab V3+ with MobileNet V2	0.86 (±0.01)	0.84 (±0.009)	0.85 (±0.008)	0.74 (±0.01)

**Table 8:** Segmentation results using validation set with k-fold on CCT, including Precision (PR), Recall (RE), Dice-score (DS), and mean IoU (mIoU).

Architecture	PR	RE	DS	mIoU
Our method	0.95 (±0.008)	0.95 (±0.003)	0.95 (±0.003)	0.91 (±0.008)
Fully Convolution Network with VGG-16	0.81 (±0.1)	0.89 (±0.1)	0.75 (±0.2)	0.73 (±0.05)
DeepLab V3+ with MobileNet V2	0.84 (±0.02)	0.87(±0.02)	0.84(±0.02)	0.73 (±0.04)

**Table 9:** Segmentation results using testing set CCT, including Precision (PR), Recall (RE), Dice-score (DS), and mean IoU (mIoU).

Architecture	PR	RE	DS	mIoU
Our method	0.96 (±0.006)	0.96 (±0.001)	0.96 (±0.001)	0.93 (±0.001)
Fully Convolution Network with VGG-16	0.66 (±0.06)	0.89 (±0.08)	0.73 (±0.06)	0.63 (±0.1)
DeepLab V3+ with MobileNet V2	0.82 (±0.02)	0.84 (±0.01)	0.82 (±0.02)	0.7 (±0.03)

**Table 10:** $R^{2}$ score using validation set with k-fold on NCCT and CCT.

Algorithm	NCCT		CCT	
	Max *R*^2^	Mean *R*^2^	Max *R*^2^	Mean *R*^2^
Our method	0.987	0.962 (±0.012)	0.983	0.98 (±0.004)
Linear Regression	0.826	0.778 (±0.011)	0.93	0.92 (±0.03)

## Data Availability

The data used for the research were obtained from the hospitals as described above. Data use was approved by the relevant institutional review boards. The data are not publicly available, and restrictions apply to their use.
